# Long-lasting control of *Anopheles arabiensis* by a single spray application of micro-encapsulated pirimiphos-methyl (Actellic® 300 CS)

**DOI:** 10.1186/1475-2875-13-37

**Published:** 2014-01-29

**Authors:** Richard M Oxborough, Jovin Kitau, Rebecca Jones, Emmanuel Feston, Johnson Matowo, Franklin W Mosha, Mark W Rowland

**Affiliations:** 1 Department of Disease Control, London School of Hygiene and Tropical Medicine (LSHTM), London, UK; 2 Department of Entomology and Parasitology, Kilimanjaro Christian Medical University College (KCMUCo) of Tumaini University, Moshi, Kilimanjaro, Tanzania; 3 Department of Entomology, Pan-African Malaria Vector Research Consortium, (PAMVERC), Moshi, Kilimanjaro, Tanzania

**Keywords:** IRS, Pirimiphos-methyl, Actellic, *Anopheles arabiensis*, Vector control, Malaria, Tanzania

## Abstract

**Background:**

Pyrethroid-resistant mosquitoes are an increasing threat to malaria vector control. The Global Plan for Insecticide Resistance Management (GPIRM) recommends rotation of non-pyrethroid insecticides for indoor residual spraying (IRS). The options from other classes are limited. The carbamate bendiocarb and the organophosphate pirimiphos-methyl (p-methyl) emulsifiable concentrate (EC) have a short residual duration of action, resulting in increased costs due to multiple spray cycles, and user fatigue. Encapsulation (CS) technology was used to extend the residual performance of p-methyl.

**Methods:**

Two novel p-methyl CS formulations were evaluated alongside the existing EC in laboratory bioassays and experimental hut trials in Tanzania between 2008-2010. Bioassays were carried out monthly on sprayed substrates of mud, concrete, plywood, and palm thatch to assess residual activity. Experimental huts were used to assess efficacy against wild free-flying *Anopheles arabiensis,* in terms of insecticide-induced mortality and blood-feeding inhibition.

**Results:**

In laboratory bioassays of *An. arabiensis* and *Culex quinquefasciatus* both CS formulations produced high rates of mortality for significantly longer than the EC formulation on all substrates. On mud, the best performing CS killed >80% of *An. arabiensis* for five months and >50% for eight months, compared with one and two months, respectively, for the EC. In monthly bioassays of experimental hut walls the EC was ineffective shortly after spraying, while the best CS formulation killed more than 80% of *An. arabiensis* for five months on mud, and seven months on concrete. In experimental huts both CS and EC formulations killed high proportions of free-flying wild *An. arabiensis* for up to 12 months after spraying. There was no significant difference between treatments. All treatments provided considerable personal protection, with blood-feeding inhibition ranging from 9-49% over time.

**Conclusions:**

The long residual performance of p-methyl CS was consistent in bioassays and experimental huts. The CS outperformed the EC in laboratory and hut bioassays but the EC longevity in huts was unexpected. Long-lasting p-methyl CS formulations should be more effective than both p-methyl EC and bendiocarb considering a single spray could be sufficient for annual malaria control. IRS with p-methyl 300 CS is a timely addition to the limited portfolio of long-lasting residual insecticides.

## Background

Indoor residual spraying (IRS) has produced profound changes in malaria burden in a range of settings with several different insecticide classes [1]. Interruption of malaria transmission in the USA was achieved partly through DDT house-spraying and led to the initiation of the World Health Organization (WHO)-led Global Malaria Eradication Scheme (1955-1969) [2]. Malaria was subsequently eradicated from Europe, parts of the Soviet Union, Israel, Lebanon, Syria, Japan, and Chinese Taiwan. Despite numerous positive outcomes, the benefits were not on the global scale that was anticipated. There were about 20 pilot IRS projects in sub-Saharan Africa between the mid 1950s and early 1960s [3] that demonstrated IRS significantly reduced malaria transmission even in highly endemic (intense transmission) areas [4]. Despite this, Africa was largely sidelined for eradication due to the high malaria burden; while elsewhere dramatic reversals were seen once IRS spraying was prematurely reduced in countries such as India and Sri Lanka [5,6]. As a result interest in IRS subsequently waned and was not taken to scale in most sub-Saharan malaria-endemic countries as part of the global eradication campaign [4,7].

Southern Africa was the exception. IRS programmes using DDT began in the 1960s and were supported for several decades, with later introduction of pyrethroids and carbamates. Countries with sustained IRS activities in Africa, including South Africa, Zambia, Namibia, Swaziland, Zimbabwe, and Botswana, achieved sizeable reductions in malaria vector populations and malaria incidence [7]. Focal IRS in the southern Africa region has remained important in areas of higher malaria burden and at risk of epidemics. In 2007, about 14 million people in southern Africa were protected by IRS [4,7].

In 2006 WHO reaffirmed the importance of IRS as a primary intervention for reducing or interrupting malaria transmission [8,9]. In recent years an unprecedented level of funding has initiated new IRS campaigns across sub-Saharan Africa, often in parallel with long-lasting, insecticide-treated bed nets (LLIN) distribution. In 2012 President’s Malaria Initiative (PMI) supported IRS in 15 African countries, covering seven million structures [10]. The implementation of new IRS programmes, together with sustained IRS programmes in Southern Africa has elevated the importance of IRS as a primary intervention for malaria control in Africa. Greater emphasis has been placed on ensuring that IRS in Africa can be sustained [11].

Pyrethroids are the only group of insecticides approved by WHO Pesticide Evaluation Scheme (WHOPES) for LLINs [12]. Pyrethroid insecticides have also been preferred for IRS in Africa in recent years due to low cost, longevity of three to six months, and low mammalian and non-target toxicity [13]. Subsequently, pyrethroid resistance has become widespread in malaria vectors across Africa [14]. Reduced efficacy of insecticide interventions in areas of pyrethroid resistant malaria vectors has been demonstrated in several settings. A notable example is in South Africa where four years after the introduction of deltamethrin IRS a four-fold increase in malaria cases was recorded in Kwa-Zulu Natal, coinciding with re-invasion of pyrethroid resistant *Anopheles funestus s.s.* This trend was reversed after re-introduction of IRS with DDT in 2000 and new introduction of artemisinin-based combination therapy in 2001, with an accompanied decline in malaria cases by 91% [15]. In Bioko Island, Equatorial Guinea a single spray round with pyrethroid failed to reduce the population density of pyrethroid-resistant *Anopheles gambiae* s.s. Subsequent spraying of a carbamate significantly reduced the number of *An. gambiae s.s.* caught exiting in window traps, thus demonstrating the utility of non-pyrethroid IRS [16].

The residual lifespan of alternative IRS insecticides is of key importance. Based on WHOPES recommendation, DDT is the longest lasting IRS, with a duration of effective action greater than six months [17]. However, the Stockholm Convention on Persistent Organic Pollutants stipulates that, ‘countries using DDT are encouraged to reduce and eliminate the use of DDT over time and switch to alternative insecticides’ [18]. Carbamates are a commonly used alternative to DDT and pyrethroids, and were sprayed in ten African countries in 2012 through PMI funding. Based on WHOPES recommendation, bendiocarb has a short residual action of only two to six months [17]. In areas of intense year-round (perennial) transmission, multiple spray rounds of short lasting insecticides are expensive, logistically demanding, and inconvenient to householders [8]. Despite added impetus for the development of new public health insecticides, notably from Innovative Vector Control Consortium (IVCC), alternative classes of insecticide for public health use are emerging slowly [11]. For improved cost-effectiveness of IRS programmes it is important to develop new long-lasting formulations of currently available insecticides [19].

Encapsulation technology can extend the residual performance of established insecticides. Pirimiphos-methyl (p-methyl) is an organophosphate insecticide, most commonly and intensively used in the protection of cereal grain [20]. Several small and medium scale IRS trials conducted since the 1970s showed high toxicity to anopheline mosquitoes [21], leading to WHOPES’ recommendation. According to WHOPES, p-methyl EC formulation has a relatively short residual IRS activity of two to three months but was used successfully for IRS in Malawi and Zambia in 2012 [22]. The overall aim of this study was to evaluate longevity of two capsule suspension (CS) formulations in comparison with emulsifiable concentrate (EC).

## Methods

### Insecticide formulations

Two capsule suspension (CS) formulation variants of Actellic 300CS, containing 300 g/L p-methyl and coded as CS ‘B’ and CS ‘BM’ (Syngenta, Basel, Switzerland) were evaluated alongside the existing EC formulation (Actellic 50EC®, Syngenta, Basel, Switzerland) in laboratory bioassays and experimental hut trials at 1 g/m^2^. Lambdacyhalothrin CS (0.03 g/m^2^) (Icon CS®, Syngenta, Basel, Switzerland) is a WHOPES recommended formulation that was sprayed in Tanzania as part of the national malaria control programme (NMCP) from 2007-2012 [23] and was included in laboratory bioassays as a positive control but was not sprayed in experimental huts (due to availability of huts).

### Laboratory assessment of residual performance

Cone bioassays to assess insecticidal duration on sprayed mud, concrete and plywood substrates were conducted every month based on WHO guidelines [9]. Substrates were stored at ambient temperature and humidity (~20-28°C, 40-80% RH). For each formulation three blocks were sprayed and ~ nine replicates of ~ ten female *Anopheles arabiensis* were tested, (i.e. three replicates per block), for an exposure of 60 minutes. This is longer than the 30 minutes standard exposure time as specified by WHO for IRS cone bioassay, regardless of the insecticide [9]. Test mosquitoes were transferred to 150 ml paper cups with 10% glucose solution provided *ad libitum* and mortality recorded after 24 hours. Substrates were sprayed at an application rate of 40 ml/sq m using a Potter Tower Precision Sprayer (Burkard Scientific, Uxbridge, UK). Resistance status of insectary-reared female test mosquitoes *An. arabiensis* Dondotha, *Culex quinquefasciatus* TPRI and *Cx. quinquefascaistus* Muheza was determined in WHO susceptibility tests (Table 1).

**Table 1:** Resistance status of insectary-reared mosquitoes to pyrethroid and organophosphate insecticides

		**% Mortality (n)**	
**Species**	**Strain**	**Lambdacyhalothrin 0.05%**	**Malathion 5%**
*Anopheles arabiensis*	Dondotha	100 (100)	100 (100)
*Culex quinquefasciatus*	TPRI	97 (208)	99 (200)
*Culex quinquefasciatus*	Muheza	35 (105)	100 (200)

Results of susceptibility testing with insectary strains exposed for one hour using WHO diagnostic dosages in cylinder bioassays.

### Indoor residual spraying experimental hut trials

An experimental hut trial was conducted at Kilimanjaro Christian Medical University College (KCMUCo) Field Station in Lower Moshi Rice Irrigation Zone (3°22’S, 37°19’E) nightly for 12 months between December 2008 and December 2009. The walls and ceiling of the p-methyl EC hut were covered with untreated plastic sheeting for 1 month in January 2010 to investigate the possibility of mosquito movement between huts. To determine the relative contribution of the sprayed mud and concrete walls to mortality of *An. arabiensis* the palm thatch ceiling was covered with unsprayed plastic sheeting every second week for 2 months from March-April 2010 in all huts. Further description of the supplementary experimental hut tests is included in the results section. *Anopheles arabiensis* densities were heavily dependent on rice cropping cycles with flooded rice fields adjacent to the Field Station being the main breeding site. In 2009, wild *An. arabiensis* were tested in WHO cylinder bioassays and were found to be susceptible to organophosphates, including p-methyl, and resistant to permethrin (Table 2).

**Table 2:** **Resistance status of wild *Anopheles arabiensis *** **to pyrethroid and organophosphate insecticides**

**Insecticide**	**Dosage (%)**	**Number tested**	**Mortality (%)**
P-methyl	0.025	40	98
P-methyl	0.05	40	100
P-methyl	0.25	40	100
Malathion	5	201	100
Permethrin	0.75	111	90

Two- to five-day old sugar-fed offspring (F1) of *Anopheles arabiensis* collected from cattle-sheds in Lower Moshi were exposed for one hour in WHO cylinders lined with papers treated with diagnostic dosages of malathion and permethrin, and a range of dosages of p-methyl.

Verandah experimental huts were constructed to a design described by WHO [9]. The working principle of these huts has been described previously [24]. The interior walls of experimental huts were plastered with either mud or concrete. A palm thatched mat, typical of organic fibres used in some rural housing [25], was affixed to the wooden ceiling before spraying.

The walls and ceiling were sprayed at an application rate of 40 ml/sq m with a Hudson X-pert sprayer (H D Hudson Manufacturing Company, Chicago, Ill, USA) with flat fan 8002E nozzle [26]. A constant flow valve (CFV) was not used, but compression was maintained at 55 psi by repressurizing after each swath. Flow rate was 840 ml/minute. A guidance pole was used to ensure a consistent vertical swath 71 cm wide and swath boundaries were marked out with chalk on walls and ceiling to improve spray accuracy. High performance liquid chromatography (HPLC) was not done to confirm the accuracy of the spray concentration. Verandahs were protected during spraying by blocking the open eaves with a double layer of plastic and Hessian sackcloth. IRS treatments were randomly assigned to huts. Rotation of IRS treatments was not feasible as the mud and concrete substrates were permanent. Hut position is known to bias the number of mosquitoes entering a hut, but is unlikely to affect the primary proportional outcomes, per cent mortality and per cent blood-fed of those entering the huts. The following treatments were sprayed in a total of six experimental huts.

● Pirimiphos methyl CS ‘B’, 1 g/sq m (one mud and one concrete walled hut)

● Pirimiphos methyl CS ‘BM’, 1 g/sq m (one mud and one concrete walled hut)

● Pirimiphos methyl EC, 1 g/sq m (one mud walled hut)

● Unsprayed (one mud walled hut)

The trial protocols were based on WHOPES procedures for small-scale field trials for IRS [9]. Adult trial participants gave informed consent and were offered free medical services during the trial and up to three weeks after the end of participation. An adult volunteer slept in each hut nightly from 20:30-06:30. Sleepers were rotated between huts on successive nights to reduce any bias due to differences in individual attractiveness to mosquitoes. Each morning mosquitoes were collected from the verandahs and window traps of huts and recorded as blood-fed or unfed and dead or alive. Live mosquitoes in the sprayed room were not collected in order to allow for natural resting times on treated surfaces, and were only collected after exiting to verandahs or window traps. 10% glucose pads were placed in the window traps and verandahs to prevent death by starvation. Live mosquitoes were transferred to 150 ml paper cups and provided with 10% glucose solution before scoring delayed mortality after 24 hours. All members of the *An. gambiae* species complex identified by morphological characteristics were assumed to be *An. arabiensis* based on recent PCR identification [27].

### Experimental hut bioassays

Cone bioassays of the sprayed walls and ceiling were conducted monthly using sugar-fed, two to five day-old female *An. arabiensis* dondotha, for an exposure of 60 minutes. In each experimental hut four to eight replicates of ten female mosquitoes were tested on the walls and ceiling surfaces. Cones were positioned randomly for each test.

### Fumigant activity

The possibility of fumigant activity of the treatments was determined using insectary-reared wild female F1 *An. arabiensis* (no tarsal contact) [9]. Wire cages measuring 15 cm × 10 cm × 10 cm covered with netting were hung in the corner of the room ~5 cm from the wall and 25 mosquitoes exposed overnight. Testing was done monthly in for all treatments until mortality decreased to low levels.

### Analysis of laboratory assessment of residual performance

Treatments were compared according to the time interval since spray application for mortality to fall to 80% (based on WHOPES criteria) and 50% [9]. Mixed effect logistic regression models were used to fit mortality trajectories over time separately for each strain of mosquito (*An. arabiensis* Dondotha, *Cx. quinquefasciatus* TPRI and *Cx. quinquefasciatus* Muheza), treatment (P-methyl EC, CS ‘B’ and CS ‘BM’ and lambdacyhalothrin CS) and substrate (mud, concrete and plywood). All statistical modelling was performed on the log odds scale at the individual mosquito level and results back transformed to the proportion scale. Linear, quadratic and cubic terms in time were specified as predictors in the models to allow for potential drops and then levelling off in mortality rates over time. A random effect was specified in all models to account for similarities in mosquitoes tested at the same time point and for potential behavioural clustering within the same test batch. The cubic equations given by the estimates from the polynomial models were solved to obtain estimates of the time points at which mortality fell to 80 and 50%. Ninety-five per cent confidence intervals (CI) were estimated using the bias corrected bootstrap method with 2,000 replications. Differences between treatments in estimated time for mortality to fall to 80 and 50% were calculated and statistically significant differences inferred from the bootstrap 95% CI (p = 0.05).

### Analysis of experimental hut bioassays

Analysis of hut bioassays was similar to that described for laboratory bioassays. For wall assays, separate models were fitted for each hut. For ceiling assays, data from huts treated with the same insecticide (but with different wall materials) were combined. There was little evidence of a departure from a linear decrease in the log odds of mortality over time for either the wall or ceiling assays, so a linear term in time was specified as the only predictor in all models.

### Analysis of experimental hut trial

The number of mosquitoes collected from the two closed verandahs was multiplied by two to adjust for the unrecorded escapes through the two open verandahs which were left unscreened to allow routes for entry of wild mosquitoes via the gaps under the eaves [9,24]. The data were analysed to show the effect of each treatment in terms of:

*Overall mortality* = Total proportion of mosquitoes dead on the morning of collection, plus delayed mortality after holding for a total of 24 hours;

*Blood feeding inhibition =* Percentage of blood-fed mosquitoes from a treated hut relative to percentage from the unsprayed negative control;

*Mortality-feeding index* = The null hypothesis was that mortality and blood-feeding are independent so that mosquitoes surviving or killed by the treatment have an equal probability of having fed or not. Deviation from the null hypothesis shows whether there is association between feeding and mortality and may indicate the sequence of events experienced by individual mosquitoes after entering in the hut. The mortality-feeding index is calculated as follows:

$$\begin{array}{l} \mathrm{Mortality}-\mathrm{feeding}\;\mathrm{index}=\left(\mathrm{total}\;\mathrm{blood}-\mathrm{fed}\;\mathrm{dead}/\mathrm{total}\;\mathrm{blood}-\mathrm{fed}\right)–\left(\mathrm{total}\;\mathrm{unfed}\;\mathrm{dead}/\mathrm{total}\;\mathrm{unfed}\right)\\ \mathrm{Interpretation}\;\mathrm{of}\;\mathrm{mortality}-\mathrm{feeding}\;\mathrm{index}\\ 0=\mathrm{equal}\;\mathrm{chance}\;\mathrm{of}\;\mathrm{unfed}\;\mathrm{and}\;\mathrm{blood}-\mathrm{fed}\;\mathrm{mosquitoes}\;\mathrm{being}\;\mathrm{killed}\\ 0\;\mathrm{to}-1=\mathrm{deviation}\;\mathrm{towards}\;\mathrm{unfed}\;\mathrm{mosquitoes}\;\mathrm{being}\;\mathrm{killed}\\ 0\;\mathrm{to}\;1=\mathrm{deviation}\;\mathrm{towards}\;\mathrm{blood}-\mathrm{fed}\;\mathrm{mosquitoes}\;\mathrm{being}\;\mathrm{killed} \end{array}$$

Separate mixed effect logistic regression models were fitted to the mortality and blood-feeding data. The main predictors in each model were treatment, one or more time parameters and interactions between treatment and each of the time terms. There was little evidence of a departure from a linear decrease in the log odds of mortality over time since spraying, so only linear terms in time were specified in the statistical model for mortality. A model with linear, quadratic and cubic terms in time provided the best fit to the blood-feeding data. A random effect was specified in both models to account for similarities among mosquitoes entering huts on the same day and potential behavioural clustering. Both models controlled for sleeper. Predicted trajectories were plotted over the duration of the 12 months for mortality alongside actual results.

## Results

### Laboratory residual bioassay

The duration of residual activity of the p-methyl formulations on mud, concrete, and plywood are presented in Table 3 and the differences in residual activity are shown in Table 4. Using >80% mortality and >50% mortality as the duration of residual efficacy, there was evidence that the two CS formulations showed significantly longer activity than the EC on mud and concrete substrates for both *An. arabiensis* and for two strains of *Cx. quinquefasciatus*, but differences between the two CS formulations were non-significant in most instances. There was no evidence that treatment performance differed between species or strains.

**Table 3:** **Estimated time (months) for mortality to decrease to 80 and 50% for *Anopheles arabiensis *, *Culex quinquefasciatus *** **TPRI and Muheza strains tested on laboratory sprayed substrates**

**Substrate**	**Insecticide**	**Estimated time to 80% mortality**		**Estimated time to 50% mortality**	
		**Time (months)**	**95% CI**	**Time (months)**	**95% CI**
***Anopheles arabiensis*** **dondotha**					
**Mud**	P-methyl EC	1.0	(0.7 to 1.8)	1.9	(1.2 to 4.2)
	**P-methyl CS B**	**4.9**	**(4.4 to 5.5)**	**7.5**	**(5.7 to †)**
	P-methyl CS BM	4.4	(3.8 to 5.1)	6.2	(5.4 to 7.0)
**Concrete**	P-methyl EC	2.3	(1.8 to 2.7)	3.1	(2.7 to 3.3)
	**P-methyl CS B**	**6.4**	**(6.1 to 6.8)**	**7.2**	**(6.9 to 7.5)**
	P-methyl CS BM	5.0	(4.4 to 5.5)	6.5	(6.0 to 7.0)
***Culex quinquefasciatus*** **TPRI**					
**Mud**	P-methyl EC	1.8	(1.4 to 2.2)	2.1	(1.7 to 2.5)
	**Lambda CS**	**2.9**	**(2.7 to 3.3)**	**3.7**	**(3.4 to 4.0)**
	P-methyl CS B	6.2	(5.3 to 7.6)	†	†
	**P-methyl CS BM**	**7.4**	**(6.8 to 8.1)**	**9.7**	**(8.6 to 11.0)**
**Concrete**	P-methyl EC	0.8	(0.7 to 0.9)	1.3	(1.2 to 1.6)
	**Lambda CS**	**5.0**	**(4.7 to 5.3)**	**5.9**	**(5.7 to 6.1)**
	P-methyl CS B	8.2	(7.5 to 9.3)	9.7	(8.9 to 10.7)
	**P-methyl CS BM**	**6.8**	**(0.6 to 7.2)**	**8.6**	**(8.1 to 9.1)**
***Culex quinquefasciatus*** **Muheza**					
**Mud**	P-methyl EC	0.8	(0.5 to 1.1)	1.3	(1.0 to 1.6)
	**Lambda CS**	**†**	**†**	**0.9**	**(0.5 to 1.4)**
	P-methyl CS B	4.0	(3.5 to 4.6)	7.1	(5.5 to 11.0)
	**P-methyl CS BM**	**3.8**	**(3.3 to 4.3)**	**6.4**	**(5.7 to 7.3)**
**Concrete**	P-methyl EC	1.0	(0.8 to 1.2)	1.4	(1.0 to 1.7)
	**Lambda CS**	**1.1**	**(0.8 to 1.6)**	**1.8**	**(1.5 to 2.2)**
	P-methyl CS B	4.9	(4.2 to 5.6)	6.5	(5.8 to 7.4)
	**P-methyl CS BM**	**4.3**	**(4.1 to 4.6)**	**5.7**	**(5.3 to 6.1)**

†Indicates that statistical models produced estimates outside the study period: for *Culex quinquefasciatus* TPRI, estimated mortality for Actellic CS-B on mud was higher than 50% throughout the entire study period; for *Culex quinquefasciatus* Muheza, estimated mortality for Lambda CS was lower than 80% throughout.

**Table 4:** Between treatment differences in estimated time for mortality to fall to 80 and 50% for mosquitoes tested on insecticide-treated substrates

**Substrate**	**Treatment comparison**	**Difference in estimated time to 80% mortality**			**Difference in estimated time to 50% mortality**		
		**Time months**	**95% CI**	**p**	**Time months**	**95% CI**	**p**
***Anopheles arabiensis*** **dondotha**							
**Mud**	CS B vs EC	3.9	(3.1 to 4.6)	<0.05	5.6	(3.0 to 12.9)	<0.05
	**CS BM vs EC**	**3.5**	**(2.6 to 4.3)**	**<0.05**	**4.2**	**(2.0 to 5.4)**	**<0.05**
	CS B vs CS BM	0.4	(-0.4 to 1.3)	n/s	1.3	(-0.7 to 11.7)	n/s
**Concrete**	CS B vs EC	4.1	(3.6 to 4.7)	<0.05	4.1	(3.7 to 4.6)	<0.05
	**CS BM vs EC**	**2.6**	**(1.9 to 3.4)**	**<0.05**	**3.4**	**(2.8 to 4.0)**	**<0.05**
	CS B vs CS BM	1.5	(0.8 to 2.2)	<0.05	0.7	(0.1 to 1.3)	<0.05
***Culex quinquefasciatus*** **TPRI**							
**Mud**	CS B vs EC	4.4	(3.4 to 5.8)	<0.05	†	†	†
	**CS BM vs EC**	**5.6**	**(4.8 to 6.3)**	**<0.05**	**7.5**	**(6.4 to 8.9)**	**<0.05**
	Lambda vs EC	1.2	(0.6 to 1.7)	<0.05	1.6	(1.0 to 2.1)	<0.05
	**CS B vs Lambda**	**3.2**	**(2.2 to 4.6)**	**<0.05**	**†**	**†**	**†**
	CS BM vs Lambda	4.4	(3.8 to 5.2)	<0.05	6.0	(4.9 to 7.4)	<0.05
	**CS B vs CS BM**	**-1.2**	**(-2.4 to 0.4)**	**n/s**	**†**	**†**	**†**
**Concrete**	CS B vs EC	7.4	(6.7 to 8.4)	<0.05	8.4	(7.5 to 9.4)	<0.05
	**CS BM vs EC**	**6.0**	**(-0.2 to 6.4)**	**n/s**	**7.2**	**(6.7 to 7.8)**	**<0.05**
	Lambda vs EC	4.2	(3.8 to 4.5)	<0.05	4.6	(4.3 to 4.9)	<0.05
	**CS B vs Lambda**	**3.2**	**(2.4 to 4.3)**	**<0.05**	**3.8**	**(2.9 to 4.8)**	**<0.05**
	CS BM vs Lambda	1.8	(-4.4 to 2.4)	n/s	2.7	(2.1 to 3.3)	<0.05
	**CS B vs CS BM**	**1.4**	**(0.5 to 7.5)**	**<0.05**	**1.2**	**(0.2 to 2.2)**	**<0.05**
***Culex quinquefasciatus*** **Muheza**							
**Mud**	CS B vs EC	3.2	(2.7 to 3.9)	<0.05	5.8	(4.2 to 9.8)	<0.05
	**CS BM vs EC**	**3.0**	**(2.5 to 3.6)**	**<0.05**	**5.1**	**(4.4 to 6.2)**	**<0.05**
	Lambda vs EC	†	†	†	-0.3	(-0.9 to 0.3)	n/s
	**CS B vs Lambda**	**†**	**†**	**†**	**6.1**	**(4.2 to 10.2)**	**<0.05**
	CS BM vs Lambda	†	†	†	5.5	(4.6 to 6.6)	<0.05
	**CS B vs CS BM**	**0.2**	**(-0.5 to 0.9)**	**n/s**	**0.7**	**(-1.2 to 4.6)**	**n/s**
**Concrete**	CS B vs EC	3.9	(3.0 to 4.6)	<0.05	5.2	(4.2 to 6.0)	<0.05
	**CS BM vs EC**	**3.3**	**(2.9 to 3.7)**	**<0.05**	**4.3**	**(3.7 to 4.8)**	**<0.05**
	Lambda vs EC	0.1	(-0.3 to 0.5)	n/s	0.4	(-0.1 to 0.9)	n/s
	**CS B vs Lambda**	**3.8**	**(3.0 to 4.6)**	**<0.05**	**4.7**	**(3.9 to 5.8)**	**<0.05**
	CS BM vs Lambda	3.2	(2.8 to 3.7)	<0.05	3.9	(3.3 to 4.4)	<0.05
	**CS B vs CS BM**	**0.6**	**(-0.2 to 1.4)**	**n/s**	**0.8**	**(0.0 to 1.9)**	**n/s**

†Indicates that statistical models produced estimates outside the study period for one or more of the treatments or their 95% CI and treatment differences cannot therefore be estimated.

**Figure 1:**
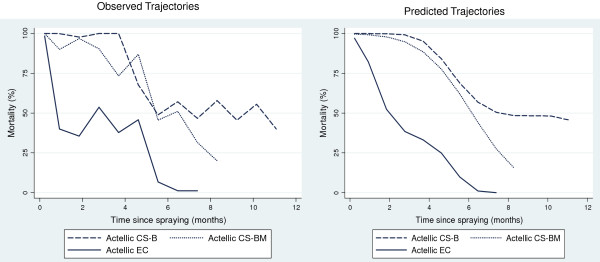
**Mortality of *Anopheles arabiensis *dondotha on mud blocks after one-hour bioassays.** Mud blocks were sprayed with p-methyl CS ‘B’, CS ‘BM’, and EC and tested at monthly intervals. Mortality for unsprayed blocks was <15% for all bioassays.

**Figure 2:**
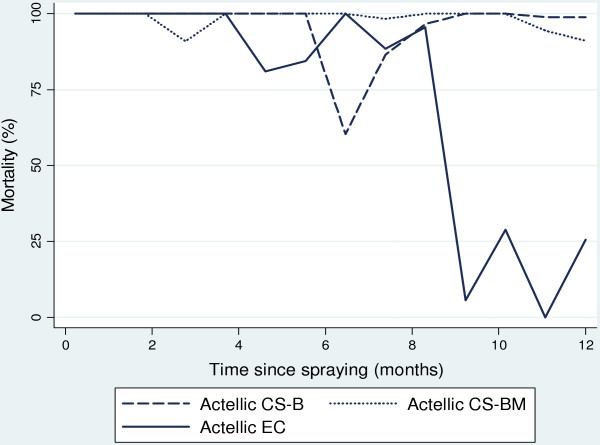
**Mortality of *Anopheles arabiensis *dondotha on plywood blocks after one-hour bioassays.** Plywood blocks were sprayed with p-methyl CS ‘B’, CS ‘BM’, and EC and tested at monthly intervals. Mortality for unsprayed blocks was <15% for all bioassays.

When sprayed on mud, the EC had a particularly short residual action against *An. arabiensis*, and killed >80% for only one month (95% CI: 0.7-1.8). CS ‘B’ and CS ‘BM’ showed substantial improvement over the EC with mortality >80% for 4.9 months (95% CI: 4.4-5.5) and 4.4 months (95% CI: 3.8-5.1) respectively (P < 0.05). The residual times for 50% mortality to be reached, (RT 50), were 7.5 months (95% CI: 5.7 to †) for CS ‘B’; 6.2 months (95% CI: 5.4-7.0) for CS ‘BM’; and 1.9 months (95% CI: 1.2-4.2) for EC (Table 3, Figure 1). On concrete CS ‘B’ produced >80% mortality for 4.1 months (95% CI: 3.6-4.7) longer than the EC against *An. arabiensis* (P < 0.05) (Table 4). Based on observed data on plywood, both CS ‘B’ and CS ‘BM’ killed >80% *An. arabiensis* for 12 months. The EC killed >80% for eight months, followed by a rapid decline to <30% after nine months (Figure 2).

### Residual activity of formulations in experimental huts

**Figure 3:**
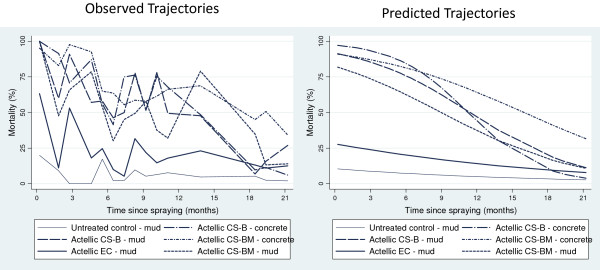
**Mortality of *Anopheles arabiensis *dondotha after one-hour bioassay on experimental hut walls.** Time after spraying is shown in months. Mortality for unsprayed walls was <15% for all bioassays.

One-hour cone bioassays of *An. arabiensis* were conducted on walls and ceilings at monthly intervals. Both CS formulations showed improvement over the EC on mud, concrete and palm thatch. Mortality was 100% one week after spraying the CS ‘B’ and CS ‘BM’ formulations on mud and concrete walls (Figure 3). Mortality was >80% for CS ‘B’ for 4.8 months (95% CI: 1.9-6.9) on mud and 7.0 months (95% CI: 5.4-8.3) on concrete, compared with 0.9 months (95% CI: 0-4.4) and 6.6 months (95% CI: 3.0-9.0) for CS ‘BM’ respectively (Table 5). The EC was ineffective on mud and killed a small proportion one week after spraying.

**Table 5:** **Estimated time (months) for mortality to decrease to 80 and 50% for *Anopheles arabiensis *** **dondotha (pyrethroid susceptible), tested on sprayed experimental hut walls (concrete and mud) and ceiling (thatch)**

**Insecticide**	**Substrate**	**Estimated time to 80% mortality**		**Estimated time to 50% mortality**	
		**Time (months)**	**95% CI**	**Time (months)**	**95% CI**
**Hut walls**					
P-methyl EC	Mud	†	†	†	†
**P-methyl CS B**	**Concrete**	**7.0**	**(5.4 to 8.3)**	**11.3**	**(10.2 to 12.4)**
	Mud	4.8	(1.9 to 6.9)	11.4	(9.9 to 13.0)
**P-methyl CS BM**	**Concrete**	**6.6**	**(3.0 to 9.0)**	**16.0**	**(13.5 to 20.6)**
	Mud	0.9	(† to 4.4)	9.0	(6.4 to 11.0)
**Hut ceilings**					
**P-methyl EC**	**Thatch**	**†**	**†**	**2.4**	**(† to 6.1)**
P-methyl CS B	Thatch	8.4	(7.4 to 9.4)	12.0	(11.2 to 12.7)
**P-methyl CS BM**	**Thatch**	**10.8**	**(9.9 to 11.7)**	**14.4**	**(13.7 to 15.2)**

†Indicates that statistical models produced estimates outside the study period: in all cases estimates were lower than the specified mortality (50 or 80%, respectively) throughout the entire study period.

**Figure 4:**
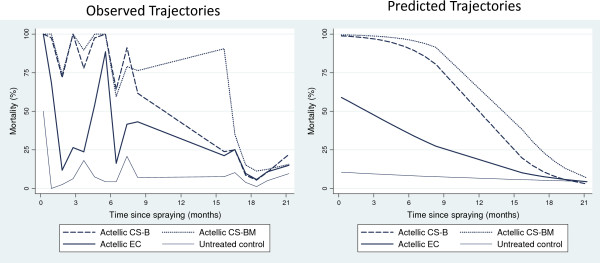
**Mortality of *Anopheles arabiensis *after one-hour bioassay on experimental hut ceiling.** One-hour cone bioassay of insectary-reared *Anopheles arabiensis* dondotha on palm thatch ceiling over time (months) after spray application.

Actellic CS on palm thatch ceiling was highly effective, with close to 100% mortality recorded for both CS formulations after six months (Figure 4) and >80% for 8.4 months for CS ‘B’ (95% CI: 7.4-9.4) and 10.8 months for CS ‘BM’ (95% CI: 9.9-11.7) (Table 5). Mortality remained high for the CS formulations and was >50% up to 12 months (95% CI: 11.2-12.7) and 14.4 (13.7-15.2) months after spraying for CS ‘B’ and ‘BM’ respectively. The EC initially killed a fairly high proportion of *An. arabiensis* but showed a marked reduction to <50% 2.4 months (95% CI: 0-6.1) after spraying.

### Twelve-months experimental hut trial against wild free-flying *Anopheles arabiensis*

**Figure 5:**
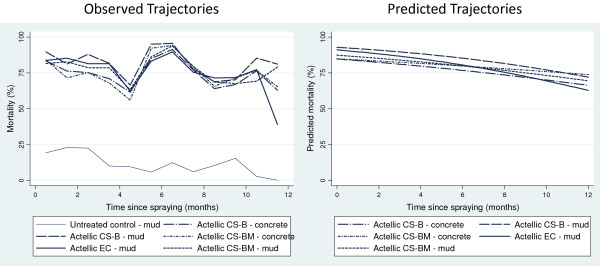
**Mortality of wild *Anopheles arabiensis *freely entering experimental huts over 12 months after spraying.** Data on wild mosquitoes recorded on a daily basis were variable. Graphs of observed mortality over time plot data pooled for each month since spraying*.*

All formulations of p-methyl (CS ‘B’, CS ‘BM’, and EC) were highly effective against free-flying wild *An. arabiensis* shortly after spray application (Figure 5). Mortality gradually decreased over time for all formulations up to five months after spraying, followed by a small increase between months five to seven, possibly due to climatic changes. Subsequently, between months seven to 12 there was a gradual decrease in mortality (Figure 5). Overall mortality rates remained high for both CS treatments up to12 months after spraying regardless of wall substrate. P-methyl EC performed equally well as CS ‘B’ and CS ‘BM’ after 12 months, based on 95% CIs from estimated curves. Twelve months after spraying predicted mortality was 62.8% (95% CI: 54.4-71.2) for EC, 72.0% (95% CI: 64.5-79.6) for CS ‘B’ (mud) and 69.5% (95% CI: 62.0-77.0) for CS ‘BM’ (mud) (Table 6).

**Table 6:** Estimated mortality (%) three, six, nine and twelve months after spraying for wild mosquitoes collected in insecticide treated huts

		**% Mortality (95% CI)**			
**Insecticide**	**Substrate**	**3 months**	**6 months**	**9 months**	**12 months**
P-methyl EC	Mud	86.6	80.5	72.5	62.8
		(83.9 to 89.4)	(77.8 to 83.3)	(67.9 to 77.2)	(54.4 to 71.2)
P-methyl CS B	**Concrete**	**81.0**	**76.8**	**71.8**	**66.3**
		**(77.7 to 84.4)**	**(73.7 to 79.8)**	**(67.1 to 76.6)**	**(58.3 to 74.3)**
	Mud	89.6	85.3	79.4	72.0
		(87.3 to 92.0)	(82.9 to 87.6)	(75.4 to 83.4)	(64.5 to 79.6)
P-methyl CS BM	**Concrete**	**82.5**	**79.8**	**76.9**	**73.8**
		**(79.3 to 85.6)**	**(77.1 to 82.6)**	**(72.9 to 81.0)**	**(67.0 to 80.5)**
	Mud	83.9	79.8	75.0	69.5
		(80.9 to 86.9)	(77.1 to 82.6)	(70.6 to 79.4)	(62.0 to 77.0)

Estimates are adjusted for sleeper and account for similarities among mosquitoes entering huts on the same day and potential behavioural clustering.

**Figure 6:**
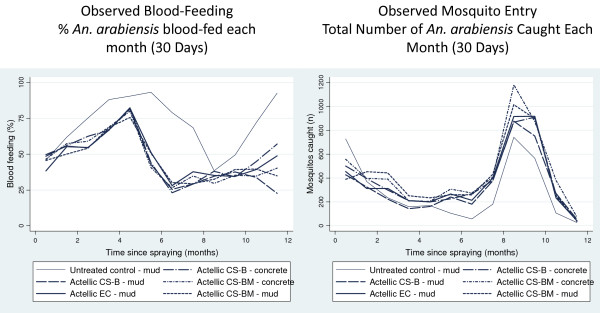
**Percentage blood-fed *Anopheles arabiensis *collected in experimental huts over time by treatment (left) and number of *Anopheles arabiensis *caught per treatment over time (right).** Data on wild mosquitoes recorded on a daily basis were variable. Graphs of blood feeding and number of mosquitoes caught over time plot data pooled for each month since spraying.

Blood-feeding was high in the unsprayed hut throughout the study but did show considerable variation over time and ranged from 40% (after nine months) to 90% (five and 12 months) (Figure 6). The two periods of lowest percentage blood feeding in the unsprayed hut, one and nine months after spraying, coincided with the period of highest mosquito density during rice transplantation cycles (Figure 6). For the first month after spraying, treated huts provided no protection from being bitten by host-seeking *An. arabiensis*. Between two and 12 months after spraying all treatments provided some degree of personal protection (Figure 6). Blood-feeding inhibition was relatively high after six and nine months across all treatments ranging between 39-49% for CS formulations and 36-43% for EC (Table 7). Blood-feeding inhibition was similar for both CS and EC formulations over the trial. The mortality-feeding index (total blood-fed dead/total blood-fed) – (total unfed dead/total unfed) was 0.08 and 0.05 for CS ‘B’ and 0.08 and 0.03 for CS ‘BM’ on concrete and mud walled huts compared with 0.07 for EC and 0.15 for the unsprayed hut (mud walls). For all treatments the mortality-feeding index was close to 0 indicating mosquitoes had an equal chance of surviving whether fed or unfed.

**Table 7:** Estimated blood feeding (%) three, six, nine and twelve months after spraying for wild mosquitoes collected in insecticide treated huts

**Insecticide**	**Substrate**	**3 months**		**6 months**		**9 months**		**12 months**	
		**Blood fed (%)**	**BFI (%)**	**Blood fed (%)**	**BFI (%)**	**Blood fed (%)**	**BFI (%)**	**Blood fed (%)**	**BFI (%)**
		**(95% CI)**		**(95% CI)**		**(95% CI)**		**(95% CI)**	
Untreated control	Mud	90	-	81	–	57	–	93	–
		(87 to 93)		(77 to 85)		(52 to 63)		(86 to 100)	
P-methyl EC	Mud	69	24	52	36	33	43	67	29
		(64 to 74)		(47 to 57)		(28 to 37)		(49 to 84)	
P-methyl CS B	Concrete	71	22	49	40	32	44	84	9
		(66 to 76)		(44 to 54)		(28 to 37)		(73 to 96)	
	Mud	66	26	50	39	31	46	47	49
		(61 to 72)		(44 to 55)		(26 to 35)		(26 to 69)	
P-methyl CS BM	Concrete	68	24	48	41	29	49	63	33
		(63 to 73)		(43 to 53)		(25 to 33)		(45 to 81)	
	Mud	67	26	49	39	31	46	63	32
		(61 to 72)		(44 to 54)		(27 to 35)		(44 to 82)	

Estimates are adjusted for sleeper and account for similarities among mosquitoes entering huts on the same day and potential behavioural clustering. BFI = blood-feeding inhibition compared to the untreated control.

Fumigant activity tested in small cages resulted in 100% mortality of *An. arabiensis* F1 one week and two months after spraying for CS ‘B’, ‘BM’ and EC formulations. A large decrease to 42% fumigant mortality was recorded after three months for CS ‘BM’ (concrete) with fumigant mortality less than 10% for all other treatments.

### Supplementary explanatory bioassays in experimental huts

The walls and ceiling of the p-methyl EC hut were covered with untreated plastic sheeting between months 12-13. This was done to investigate the possibility of mosquito movement between huts*,* picking up a lethal dosage of p-methyl CS before exiting, flying into the EC hut and dying. All other huts were left uncovered. Mortality for the covered EC hut was 29%, which was greater than the unsprayed hut, 1% (P = 0.001) but less than huts sprayed with CS ‘B’, 65%, 78% and CS ‘BM’, 67%, 74% with concrete and mud walls respectively (P = 0.001) (Table 8). The proportion of *An. arabiensis* that blood-fed was significantly higher in the covered EC hut (63%), than for CS formulations (19-38%, P < 0.05) but was less than the unsprayed hut 94% (P = 0.001).

**Table 8:** Supplementary experimental hut results for percentage mortality and blood-feeding, 13-16 months after spraying

**Time after spraying**	**Outcome measures**	**Untreated (Mud)**	**CS-B (Concrete)**	**CS-BM (Concrete)**	**CS-B (Mud)**	**CS-BM (Mud)**	**EC (Mud)**
13 Months (EC Walls & Ceiling Covered)	Total Caught	92	181	204	143	170	115
	**% Mortality**	**1**	**65**	**67**	**78**	**74**	**29**
	95% CI	(1 to 6)	(51 to 77)	(45-83)	(63-88)	(61-83)	(13-51)
	**% Blood-fed**	**94**	**32**	**30**	**19**	**38**	**63**
	% BFI	-	66	68	80	60	33
15-16 Months (Ceiling Uncovered)	Total Caught	411	592	870	576	685	629
	**% Mortality**	**5**	**34**	**42**	**48**	**63**	**43**
	95% CI	(2-12)	(27-42)	(33-51)	(36-59)	(46-77)	(31-55)
	**% Blood-fed**	**59**	**48**	**53**	**51**	**42**	**52**
	% BFI	-	19	10	14	29	12
15-16 Months (Ceiling Covered)	Total Caught	303	557	455	390	498	580
	**% Mortality**	**7**	**48**	**49**	**49**	**53**	**46**
	95% CI	(3-15)	(41-55)	(38-60)	(38-59)	(41-64)	(37-55)
	**% Blood-fed**	**69**	**47**	**46**	**51**	**45**	**54**
	% BFI	-	32	33	26	35	22

During month 13 the walls and ceiling of the hut sprayed with p-methyl EC were covered with plastic sheeting. Between months 15 and 16 the treated walls of every hut were covered with plastic sheeting for one out of every two weeks. Data are grouped according to whether the walls were covered or uncovered. BFI = blood-feeding inhibition compared to untreated control.

To determine the relative contribution of the sprayed mud and concrete walls to mortality of *An. arabiensis* the palm thatch ceiling was covered with unsprayed plastic sheeting every second week between months 15-16. As the palm thatch ceiling remained highly insecticidal over the duration of the study (Figure 4) the hypothesis was that it masked any differences in efficacy between the concrete and mud walls (Figure 3). The covering of the ceiling had little impact on overall mortality trends for the EC hut (mud) with 43% mortality when uncovered and 46% covered (P = 0.255) (Table 8). For both CS ‘B’ and CS ‘BM’ any differences in mortality after covering the ceiling were small for both mud and concrete huts.

**Figure 7:**
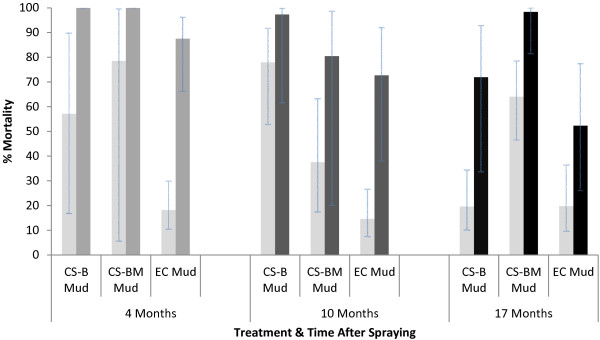
**Results of extended duration bioassays on walls of experimental huts.** Percentage mortality of insectary-reared *Anopheles arabiensis* dondotha following cone bioassay with standard exposure time of one hour (light bars) and extended exposure (darker bars) of two hours (four months), four hours (ten months), 12 hours (17 months) on sprayed mud walls. Mortality for unsprayed walls was <20% for all bioassays.

Extended cone bioassays of up to 12 hours were undertaken, as may occur when mosquitoes enter a house early in the evening to blood-feed and subsequently rest on treated surfaces until the following morning before exiting. With one-hour exposure, four months after spraying the CS ‘B’ and CS ‘BM’ killed a far greater proportion (P = 0.001) of *An. arabiensis* than EC, with mortality 18% for EC compared with 57% and 79% for CS ‘B’ and CS ‘BM’ (Figure 7). With longer exposure of two hours, the EC killed 88% of *An. arabiensis* compared with 100% for CS formulations. A similar trend was observed after ten months as the EC killed 15% with one-hour exposure but killed 73% with a four-hour exposure compared with 80% for CS ‘BM’ (P = 0.401) and 97% for CS ‘B’ (P = 0.014). After 17 months mortality was low for both CS ‘B’ (20%) and EC (20%) with one-hour exposure but increased to 52% for EC, 72% CS ‘B’, and 98% for CS ‘BM’ with 12-hour exposure.

## Discussion

Laboratory bioassays showed that p-methyl CS ‘B’ and CS ‘BM’ formulations were effective at killing high proportions (>80%) of *An. arabiensis* and *Cx. quinquefasciatus* for significantly longer than the EC formulation on mud, concrete and plywood substrates. The most important improvement was observed on mud. The EC was ineffective on mud and killed >80% of *An. arabiensis* and *Cx. quinquefasciatus* for one month or less. In contrast, the best performing CS formulation killed >80% of *An. arabiensis* for five months and sustained control above 50% for longer than seven months. Similar longevity was observed in The Gambia where p-methyl CS sprayed in village houses persisted for at least five months (when testing was ended) on mud and painted walls [28]. Mud is a problematic substrate for IRS owing to loss of available insecticide due to sorption. Early work in Tanzania in the 1960s characterized the performance of organophosphates and carbamates on various types of soil and showed rapid loss of efficacy on several types of mud, while on less porous substrates, such as wood, high levels of mortality were recorded over several months [29,30]. In the present study, micro-encapsulation substantially improved the surface bioavailability of p-methyl on mud. Mud or adobe is still a common wall material in rural, low-income areas of Africa. In Tanzania in 2010, 78% of houses were constructed from a form of mud; the most common types being mud plaster (27%), sun-dried mud bricks (28%) and burnt mud bricks (23%) [25].

Both CS formulations showed improved longevity over EC on concrete and wood substrates in bioassays. The alkaline Ph of concrete can rapidly degrade insecticides commonly used for IRS, particularly pyrethroids, resulting in reduced residual efficacy [17]. In laboratory bioassays on plywood, CS formulations lasted for several months longer than the EC, and killed >80% of *An. arabiensis* 12 months after spraying. Wood is relatively non-porous with a tendency for long residual bioavailability of organophosphates and pyrethroids [29,31].

Cone bioassays on mud and concrete experimental hut walls showed similar findings to laboratory results and showed that both CS formulations were effective for significantly longer than the EC. For all bioassays in the laboratory and experimental huts an exposure time of 60 minutes was used rather than the standard WHOPES 30 minutes exposure. It is likely that the residual duration of action would be shorter if tested using WHOPES guidelines.

Results for free-flying, wild *An. arabiensis* showed that huts sprayed with p-methyl CS formulations maintained high rates of mortality for up to 12 months after spraying. This finding is comparable to that in Benin where 1 g/sq m of p-methyl sprayed in mud and concrete experimental huts killed around 75% of wild free-flying *An. gambiae s.s.* ten months after spraying [32].

In Tanzania, there was an increase in mortality for all formulations five to seven months after spraying between May-July. This was the cool season when mean night-time temperature outdoors dropped to 20°C compared with 24°C inside the experimental huts (USB Wireless Touchscreen Weather Forecaster, Maplin, UK). This may have resulted in longer indoor resting times, which would explain the increase in mortality during this three-month period. It has been reported elsewhere that at higher altitude where differences between indoor and outdoor temperature are greatest, indoor resting is more common [33,34].

An unexpected finding was that the EC formulation matched the performance of the CS against wild free-flying *An. arabiensis* despite being considered by WHOPES to have an effective duration of only two to three months [17,32]. Recent studies in Ghana on painted cement, and Mozambique on several surfaces, showed high levels of mortality for the EC formulation > four months after spraying, indicating that the EC can remain effective for a relatively long duration [35]. In this study the EC maintained high levels of mortality for wild free-flying *An. arabiensis* but paradoxically showed poor performance in one-hour cone bioassay on hut walls only weeks after spraying. Several explanations were postulated:

*Mosquito resting location*: Mortality in the EC hut may have been generated by tarsal contact with palm thatch ceiling, with mud walls providing a small proportion of overall mortality. Covering the ceiling with untreated plastic did not result in a decrease in mortality, indicating that mosquitoes were able to pick up a lethal dosage from treated mud walls.

*Mosquito movement between huts:* It was plausible that mosquitoes were picking up a lethal dosage of p-methyl CS before exiting through open verandahs, flying into the EC hut and falsely being recorded as killed by the EC. Covering all sprayed surfaces (walls and ceiling) with untreated plastic for one month (13 months after spraying) in the EC hut should have resulted in low mortality rates similar to an unsprayed hut if there was no movement of mosquitoes between huts. When covered, mortality was 29%, which although slightly higher than the unsprayed hut, suggested that few mosquitoes were flying between huts. Throughout the trial mortality in the unsprayed control was <20%. This suggests that mortality was generated by insecticidal activity within each individual hut and any movement of mosquitoes between huts had a limited effect on mortality trends.

*Mosquito resting duration:* The standard exposure time as specified by WHO for IRS cone bioassay is 30 minutes, regardless of the insecticide [9]. This exposure time is probably suitable for excito-repellent insecticides such as pyrethroids and DDT. Resting times of blood-fed *An. gambiae* on a wall sprayed with a non-irritant insecticide, such as p-methyl, may be longer than 30 minutes. For this study an exposure of one hour was selected for monthly bioassays with supplementary bioassays of up to 12 hours. In the EC hut the finding that one-hour bioassays killed a small proportion of *An. arabiensis*, while hut collections showed high levels of mortality may indicate that mosquitoes either, i) rested for a short time and exited before picking up lethal dosage or ii) rested for several hours. Extended cone bioassay of two hours after four months and four hours after ten months showed high levels of mortality for both EC and CS formulations. *Anopheles arabiensis* may have rested on treated surfaces for several hours overnight and may partially explain why EC mortality was similar to that of the CS formulations for wild, free-flying *An. arabiensis*. While this offers some understanding to why the EC was effective for a longer duration than expected, it does not provide a full explanation for this. As new insecticides are developed for IRS with low excito-repellency, WHOPES may have to revisit the standard 30 minutes exposure for IRS, if this period of exposure does not provide an accurate prediction of field performance.

The mortality-feeding index showed that unfed mosquitoes were equally likely to be killed by p-methyl as those blood-fed. The concept of IRS is to kill mosquitoes that blood-feed and then rest on treated surfaces while processing the blood meal. This finding indicates that some *An. arabiensis* rested on hut surfaces before attempting to blood-feed and explains why there was some protective effect of p-methyl IRS [36]. There were apparent seasonal changes in percentage blood-feeding in the unsprayed hut. The periods of lowest proportion blood-fed coincided with peak mosquito densities during rice transplantation. It is likely that a larger proportion of newly emerged *An. arabiensis* entered experimental huts from adjacent paddies for resting or sugar feeding, rather than host-seeking [37].

There was a fumigant effect of all formulations that killed a high proportion of mosquitoes in cage bioassays during the first two months after spraying. The microcapsules in the CS would have limited any fumigant effect because the majority of active ingredient is enclosed within the capsule membrane; however some active ingredient is also present in external solution. Slow release of active ingredient from microcapsules was sufficient for contact mortality but insufficient for a fumigant effect. Questionnaires of volunteers sleeping during the hut trial resulted in Actellic EC ranked consistently last in terms of odour appeal, with typical comments including, “Smells like cabbage and white spirit” or, “Not pleasant and produces irritation”. The CS formulations ranked better, and were generally considered to be much milder than the EC, with comments such as, “Smells like cow insecticide, appealing as not too strong”.

Of 17 African countries sprayed with PMI-funded IRS in 2012, only one was classified as having pyrethroid susceptible anophelines; the remainder had confirmed or emerging resistance [10]. The Global Plan for Insecticide Resistance Management (GPIRM) states that in areas of pyrethroid resistance IRS rotations should be used with non-pyrethroid insecticides [38]. Despite added impetus from the IVCC there have been no new insecticides for IRS and LLIN since the pyrethroids in the 1980s [11]. As a result, the majority of African PMI-funded IRS programmes are currently spraying IRS with bendiocarb which has a short residual efficacy of only two to six months and is relatively expensive [10,17]. In Malawi, where resistance to both pyrethroids and carbamates was detected, p-methyl EC was sprayed in 2011, but “although effective, the high unit cost substantially increased the IRS costs and PMI subsequently suspended direct support due to increased costs” [39]. Long-lasting p-methyl CS formulations should be more cost-effective than both p-methyl EC and bendiocarb, but this estimation is sensitive to both the duration of efficacy and the relative cost per unit area sprayed. Use of p-methyl IRS + pyrethroid LLIN is preferential for resistance management to pyrethroid IRS + pyrethroid LLINs as p-methyl and pyrethroids have different modes of action which should result in redundant killing of mosquitoes resistant to a single insecticide [40]. Cross-resistance of organophosphates and carbamates due to altered acetylcholinesterase (AChE) target site is present at low frequency in limited parts of west and central Africa and may increase in frequency as a result of current IRS programmes using bendiocarb. Nevertheless, IRS with p-methyl CS should prove an effective solution for control of pyrethroid resistant *An. gambiae* and, having received recent recommendation from WHO [41], is a welcome addition to the limited portfolio of long-lasting IRS.

### Ethical approval

Ethical approval was granted from the review boards of LSHTM (5256) and Tanzania National Institute of Medical Research (NIMR/HQ/R.8c/Vol.I/24).

## Competing interests

The authors declare that they have no competing interests.

## Authors’ contributions

RMO participated in the study design, data collection and analysis and drafted the manuscript. JK participated in the study design, data collection and helped to draft parts of the manuscript. RJ performed the statistical analysis and provided critical comments on the manuscript. EF and JM participated in data collection and was involved in drafting parts of the manuscript. FWM participated in study design and critically revised the manuscript. MWR participated in study design, analysis and multiple revisions of the manuscript. All authors read and approved the final manuscript.
